# A Case of Glomangiopericytoma at the Nasal Septum

**DOI:** 10.1007/s12105-017-0870-6

**Published:** 2017-11-22

**Authors:** Takashi Anzai, Tsuyoshi Saito, Sho Tsuyama, Miri Toh, Katsuhisa Ikeda, Shin Ito

**Affiliations:** 1 Department of Otorhinolaryngology Head and Neck Surgery, Juntendo University School of Medicine, 2-1-1 Hongo, Bunkyo-ku, Tokyo, 113-8421 Japan; 2 Department of Human Pathology, Juntendo University School of Medicine, 2-1-1 Hongo, Bunkyo-ku, Tokyo, 113-8421 Japan

**Keywords:** Glomangiopericytoma, Endoscopic sinus surgery, Nasal septum, Hemangiopericytoma, β-Catenin

## Abstract

Glomangiopericytoma (GPC) is a rare sinonasal perivascular tumor that accounts for < 0.5–1% of all sinonasal tumors. GPC is categorized as a low-grade neoplasm with borderline malignancy and a tendency of local recurrence. GPC is a rare mesenchymal neoplasm characterized by the perivascular proliferation of tumor cells, and it requires being distinguished from solitary fibrous tumors. Here, we report a case of GPC in a 68-year-old male patient who presented at the emergency room of our hospital with a complaint of sudden epistaxis. A small, reddish, protruding tumor was observed on the right nasal septum. A biopsy revealed a possible perivascular tumor such as a GPC or solitary fibrous tumor. Thus, we performed complete resection with endoscopic surgery. The size of the resected tumor was 12 × 5 mm, and it showed a uniform proliferation of oval-to-short spindle-shaped cells with slightly branching vascular structures. The tumor cells showed minimal cytologic atypia and there were an average of 3 mitoses in 10 high power fields. Necrosis was not observed. The tumor cells showed strong and diffuse nuclear immunostaining with beta catenin and were negative with STAT6, CD34 and bcl-2. The MIB-1 labeling index was approximately 5%. Genetic testing revealed *CTNNB1* mutation (p.S33C). Thus, a diagnosis of low grade GPC was made on the biopsy and the patient could be successfully treated with endoscopic resection.

## Introduction

Glomangiopericytoma (GPC) is a rare tumor arising from the pericytes surrounding the capillaries. It was named sinonasal-type hemangiopericytoma (HPC) and accounts for < 0.5–1% of all sinonasal tumors. GPC was first reported as a hemangiopericytoma in 1942; however, the World Health Organization (WHO) classified this disease as GPC in 2005. GPC is a rare mesenchymal neoplasm that exhibits the perivascular proliferation of tumor cells, and this tumor requires being distinguished from solitary fibrous tumors (SFT).

We report a case of GPC in a 68-year-old male patient who visited the emergency room of our hospital with the complaint of sudden epistaxis. We performed endoscopic surgical resection. A detailed histological examination including genetic testing revealed a GPC.

## Case Report

**Fig. 1:**
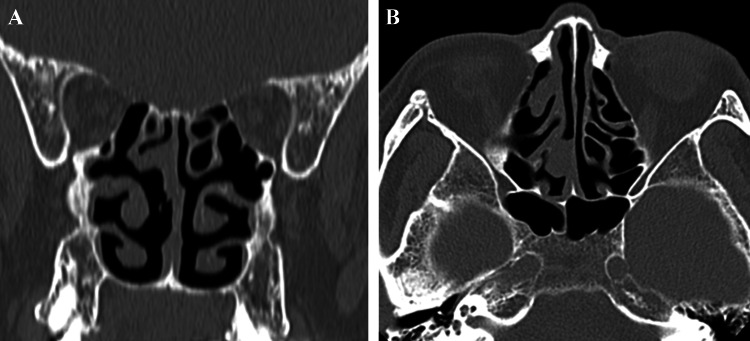
**a** and **b** A computed tomography scan showed a small mass in the right nasal cavity that developed from septal wall

**Fig. 2:**
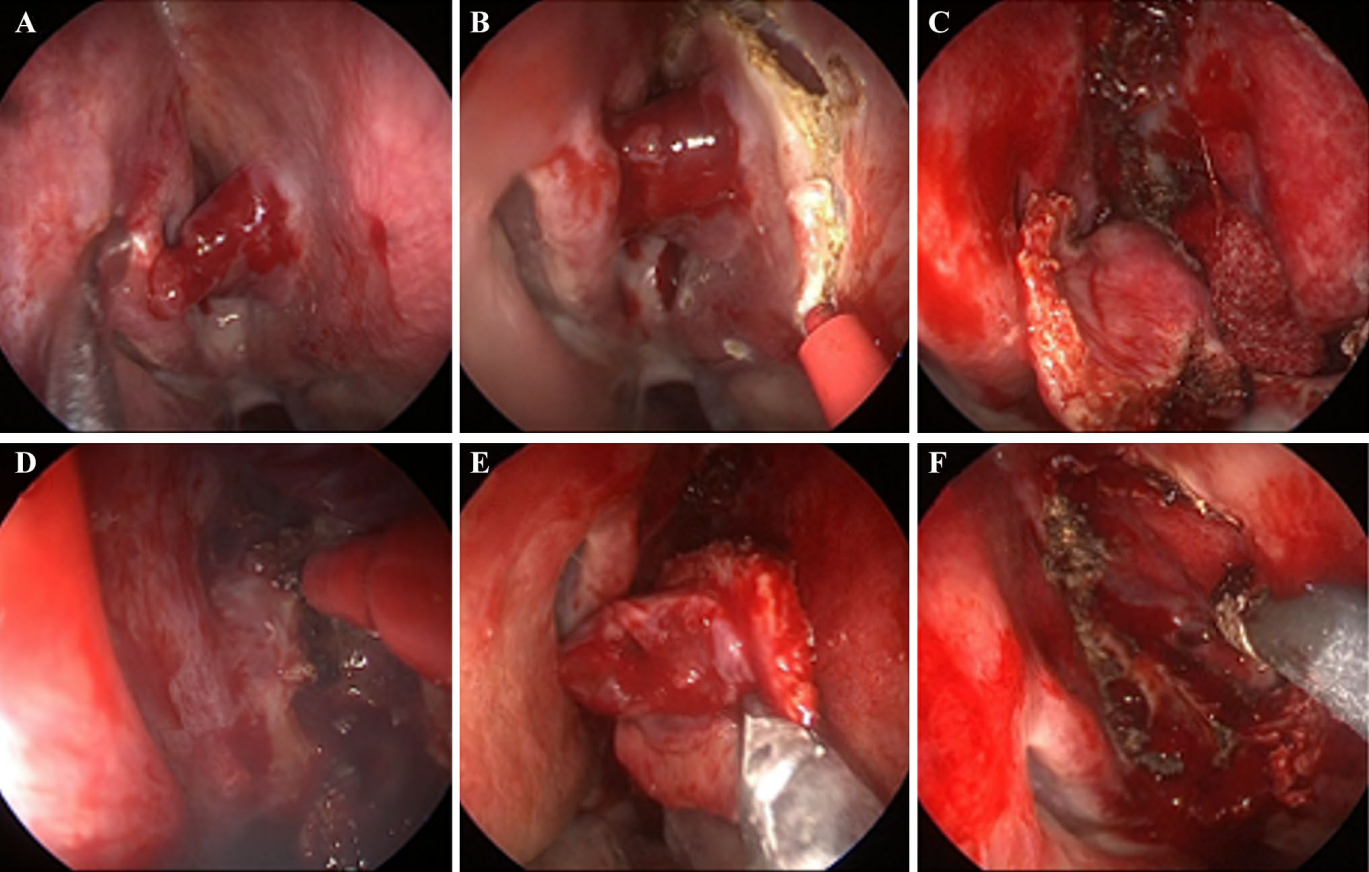
The intraoperative findings. **a** The tumor was found on the right nasal septal wall mucosa. **b** An incision was made with margins 5 mm from the tumor. **c** Resection was achieved using a Colorado Needle Scalpel. **d** Removing the tumor. **e** After removing the tumor

**Fig. 3:**
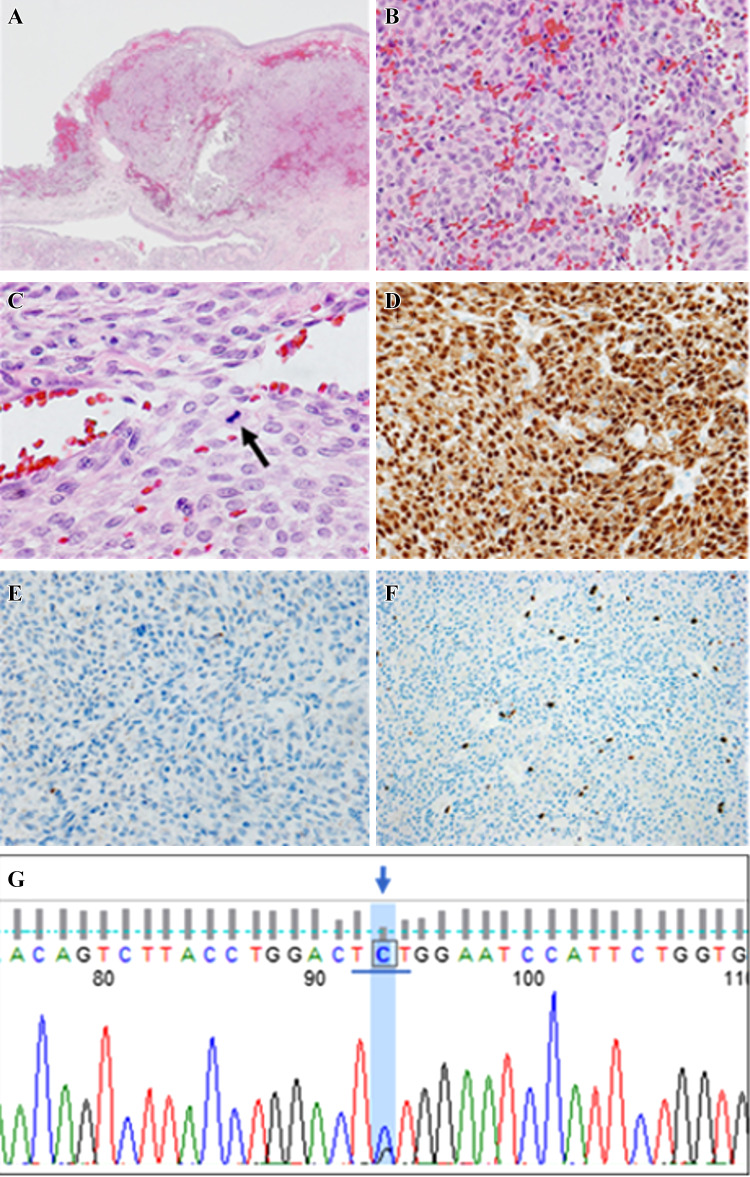
Histological and immunohistochemical features. The tumor was observed beneath the epithelium and stromal bleeding was noted (**a**). The tumor histologically showed a uniform proliferation of oval to short-spindle shaped cells with slightly branching vascular structures (**b**). The tumor cells showed less atypia and mitotic figures were less than 3/10 in high-power-fields (**C** arrow). Tumor cells were diffusely and strongly positive for β-catenin nuclear staining (**d**), but negative for STAT6 (**e**). The MIB-1 labeling index was less than 5% (**f**). Genetic testing revealed a TCT to TGT substitution at codon33, resulting in an amino acid substitution from serine to cytosine (p.S33C) (**g**)

A 68-year-old man visited the emergency room of our hospital with the complaint of sudden epistaxis. It was his first episode of epistaxis. A small reddish tumor was observed on the nasal septum using a nasal video scope. A computed tomography scan showed a small mass (about 5 mm) in the right nasal cavity that had arisen from the septal wall (Fig. 1). Although nasal packing was performed, oozing from the tumor continued. GPC was suspected based on findings of a histologic examination of the biopsy specimen, and the differential diagnosis included SFT. After 1 month, the patient was scheduled for an endonasal surgery. Pre-operative tumor embolization was not performed in light of the small size of the tumor. A reddish mass with a smooth surface extended from the high septum to the skull base. Tumor resection was designed with 5 mm margins, and resection was achieved using a Colorado Needle Scalpel (Fig. 2). Complete tumor dissection was achieved with ease. The blood loss was 10 mL, and operation time was 30 min. It was a subepithelial, well-delineated tumor, and the epithelium was partially eroded (Fig. 3a). The size of the tumor was 12 × 5 mm, and it histologically showed a uniform proliferation of oval-to-short spindle-shaped cells with slightly branching vascular structures (Fig. 3b). Stromal bleeding was also noted; however, no necrosis was observed. The tumor cells showed minimal cytologic atypia and there were an average of 3 mitoses in 10 high power fields (Fig. 3c). Tumor cells were diffusely and strongly positive for β-catenin nuclear staining (Fig. 3d), but negative for STAT6 (Fig. 3e). The MIB-1 labeling index was < 5% (Fig. 3f) and tumor cells were negative for CD34 and bcl-2. The surgical margin was negative for tumor cells. Genetic testing using DNA extracted from formalin-fixed paraffin embedded tissue revealed *CTNNB1* mutation (p.S33C) (Fig. 3g). Based on these findings, we diagnosed the patient with GPC.

## Discussion

GPC is a rare sinonasal perivascular tumor that had been designated as an HPC-like tumor or sinonasal HPC until approximately 10 years previously. Its similarity with a glomus tumor led the WHO to change this term to GPC in 2005. The etiology of GPC remains unclear. High vascularization caused by a previous trauma, hypertension, pregnancy, and prolonged use of corticosteroids are considered as predisposing factors, albeit there is no supporting evidence [1].

GPC exhibits a low malignant potential according to the WHO classification. Thompson et al. reported 17.8% of their cases with follow-up (17 of 101), ranging from a few weeks to 12 years after initial presentation [2]. The local recurrence may be a consequence of incomplete initial excision; therefore, it is advisable to consider it as a residual disease. In our case, the tumor was completely resected, the potential of recurrence and metastasis can be considered to be low. Blood control is essential for endoscopic resection because GPC is a tumor rich in vascular structures.

GPC is a rare mesenchymal neoplasm that shows the perivascular proliferation of tumor cells and requires being distinguished from SFT. SFT is characterized by chromosomal translocation resulting in the formation of *NAB2-STAT6* fusion gene. In addition to the detection of the *NAB2-STAT6* fusion gene, immunohistochemical nuclear staining of STAT6 by the translocated NAB2-STAT6 fusion protein to the nucleus is a gold marker for this tumor [3]. Immunohistochemistry findings revealed that STAT6 nuclear staining was negative in this case, disproving the histological diagnosis of SFT. A recent report demonstrated a frequent gain-of-function β-catenin mutation in 12 of 13 GPCs, proposing the nuclear accumulation of β-catenin as a diagnostic marker for this tumor [4]. Immunohistochemistry results showed strong and diffuse nuclear staining of β-catenin in this case, consistent with this finding. Furthermore, genetic testing using DNA extracted from formalin-fixed paraffin embedded tissue revealed *CTNNB1* mutation (p.S33C) that encodes β-catenin. Based on these findings, we diagnosed this tumor as GPC. The prognostic factors for aggressive behavior in such a tumor are a larger tumor size (> 5 cm), bone invasion, profound nuclear pleomorphism, increased mitotic activity (> 4/10 HPFs), necrosis, and a higher proliferative index (> 10%) [5]. None of these factors was observed in the present case; therefore, a better prognosis can be expected.

## Conclusion

We could successfully treat this patient with endoscopic surgical resection, as a minimally invasive surgery. GPC requires being distinguished from SFT. A detailed histological examination including immunohistochemical testing (STAT6 and β-catenin) and genetic testing (*CTNNBB1* mutation) can be used to diagnose a tumor as GPC.
